# Downregulation of LUZP2 Is Correlated with Poor Prognosis of Low-Grade Glioma

**DOI:** 10.1155/2020/9716720

**Published:** 2020-07-09

**Authors:** Yong Li, Gang Deng, Yangzhi Qi, Huikai Zhang, Hongxiang Jiang, Rongxin Geng, Zhang Ye, Baohui Liu, Qianxue Chen

**Affiliations:** Department of Neurosurgery, Renmin Hospital of Wuhan University, 9 Zhangzhidong Road and 238 Jiefang Road, Wuchang, Wuhan, Hubei 430060, China

## Abstract

**Background:**

LUZP2 is a protein limitedly expressed in the brain and spinal cord, while there are few studies on it in brain tumors. Low-grade glioma (LGG) is one of the most common brain tumors. However, the biological behavior of LGG is not very clear at present. This study was aimed at exploring the role of LUZP2 in LGG.

**Methods:**

By data mining in The Cancer Genome Atlas (TCGA) and Chinese Glioma Genome Atlas (CGGA), the expression, clinical characteristics, and potential regulatory mechanism of LUZP2 in LGG were assessed. The regulatory miRNAs of LUZP2 were predicted using miRDB, TargetScan, and miRTarBase. Meanwhile, the potential biological function of coexpressed genes was investigated by GO and KEGG analyses.

**Results:**

LUZP2 expression was downregulated with the increase of tumor grade (*p* < 0.05). Low LUZP2 expression independently predicted poor OS in LGG in TCGA cohort and the CGGA part B and part C cohorts (all *p* < 0.001). Additionally, LUZP2 was targeted by miR-142-5p according to 2 prediction databases and 1 validated database, which was negatively related to LUZP2 mRNA expression (*p* < 0.001). Kaplan-Meier analyses demonstrated that low miR-142-5p expression was significantly associated with poor OS (*p* < 0.001). Furthermore, coexpression genes of LUZP2 were significantly involved in nervous system development and metabolic pathways.

**Conclusions:**

LUZP2 may be crucial for nervous system extracellular matrix development and serve as an important clinical biomarker for LGG patients. miR-142-5p upregulation could be the upstream regulator that contributed to LUZP2 downregulation.

## 1. Background

Glioma, which has the characteristics of a high recurrence rate, high disability rate, and high mortality, is one of the most serious diseases of the human nervous system [1, 2]. The World Health Organization classified gliomas into 4 grades (WHO grades I, II, III, and IV) [3]. LGG usually refers to gliomas other than glioblastoma malformation (GBM). LGGs account for about 5% of all primary brain tumors and 15%-25% of all gliomas. Low-grade glioma is relatively benign and slow-growing. However, despite growing slowly initially, they usually transform to GBM with time [4, 5]. Approximately 50% of patients with LGGs will experience malignant transformation usually within 5 years [6]. However, the biological behavior of LGGs is not very clear at present.

LUZP2 (leucine zipper protein 2 gene) on Chr 11p13–11p14 encodes a leucine zipper protein, which is normally expressed only in the brain and the spinal cord. At present, we do not know much about the specific functions of LUZP2 and the articles about LUZP2 are very few. LUZP2 has been reported to be deleted in patients with Wilms' tumor-Aniridia-Genitourinary anomalies-mental Retardation (WAGR) syndrome, which is a scarce congenital anomaly syndrome consisting of Wilms' tumor, genital anomalies, aniridia, and mental retardation [7, 8]. It is reported that polymorphic variants in this gene are associated with the late-onset Alzheimer's disease and schizophrenia [9, 10]. A genome-wide association study shows that rs7943454 in LUZP2 was associated with plasma NFL with suggestive levels and rs7943454 in LUZP2 was associated with the onset risk of AD and atrophy of the right middle temporal gyrus in the whole cohort [11].

Currently, little research has been done on the relationship between LUZP2 and cancers. It is reported that LUZP2 mRNA expression is upregulated in hormone-naive prostate cancer (PC) compared with normal prostate tissues but downregulated during the development from hormone-naive PC to castration-resistant prostate cancer (CRPC) [12]. However, the expression profile of LUZP2 and its role in LGG remain unclear.

In this study, we search the expression profile and clinical significance of LUZP2 to investigate the potential mechanisms of its regulation and underlying biological function in low-grade glioma by data mining in The Cancer Genome Atlas (TCGA) and Chinese Glioma Genome Atlas (CGGA).

## 2. Materials and Methods

### 2.1. Data Processing

The LUZP2 mRNA expression level in several common cancers (including normal tissues and tumor tissues) was reviewed by using GEPIA (http://gepia.cancer-pku.cn) [13]. The gene expression profiles of GSE16011 were downloaded from the Gene Expression Omnibus database [14]. The mRNA-seq, miRNA-seq, and clinical data (level 3) of LGG patients were drawn from TCGA database (TCGA-LGG dataset) (https://cancergenome.nih.gov/). Another LGG expression and clinical dataset were downloaded from CGGA (http://cgga.org.cn/) part B and part C as the validation cohort [15–18]. All non-GBM patients with follow-up time greater than 90 days were selected. TCGA-LGG dataset included a total of 516 samples, 505 of which were selected. The CGGA part B dataset included a total of 693 samples, 414 of which were used as validation. The CGGA part C dataset included a total of 325 samples, 174 of which were selected.

### 2.2. Coexpression Analysis of mRNAs

The R package “limma” was used to find the coexpression genes of LUZP2. *p* value below 0.001 and correlation coefficient greater than 0.4 were considered statistically significant.

### 2.3. Construction of a Transcription Factor Regulatory Network

318 transcription factor genes (TFGs) were downloaded from cistrome (http://cistrome.org/). TFGs with *p* value below 0.001 and correlation coefficient greater than 0.4 were considered potential regulatory transcription factors of LUZP2 in LGG.

### 2.4. Predicting the Regulatory miRNAs of LUZP2

The regulatory miRNAs of LUZP2 were predicted using two prediction databases: miRDB v6.0 (http://www.mirdb.org/) and TargetScan v7.2 (http://www.targetscan.org/). Validated regulatory miRNAs of LUZP2 were obtained from miRTarBase v7.0 (http://carolina.imis.athena-innovation.gr/). The miRNAs simultaneously occurring in the prediction and validation cohorts were defined as the potential regulatory miRNAs of LUZP2 in LGG.

### 2.5. Functional Enrichment Analysis

We performed Gene Ontology (GO) and Kyoto Encyclopedia of Genes and Genomes (KEGG) pathway analyses of the coexpression genes by R package “enrichplot.” We used “adjusted *p* < 0.05” as the cutoff criteria to weed out the enriched GO terms and KEGG pathways.

### 2.6. Statistical Analysis

The diagnostic value of LUZP2 in LGG was judged by receiver operating characteristic (ROC) curves using area under the curve value (AUC). The LUZP2 expression level in LGG tissues was then divided into 2 groups (low and high LUZP2 expression) based on the median. Survival analysis for overall survival (OS) was performed utilizing the Kaplan-Meier method and log-rank test. All statistical analyses above were performed in R version 3.3.6 (http://www.r-project.org/). The univariate and multivariate Cox regression model was carried out to evaluate the independent factors associated with the OS. The univariate and multivariate Cox analyses were performed using SPSS 25.0. In the univariate Cox analysis, factors with *p* value below 0.1 were included in the multivariate Cox analysis. A two-tailed *p* value below 0.05 was considered statistically significant.

## 3. Results

### 3.1. LUZP2 Expression Was Downregulated with the Increase of Tumor Grade

**Figure 1:**
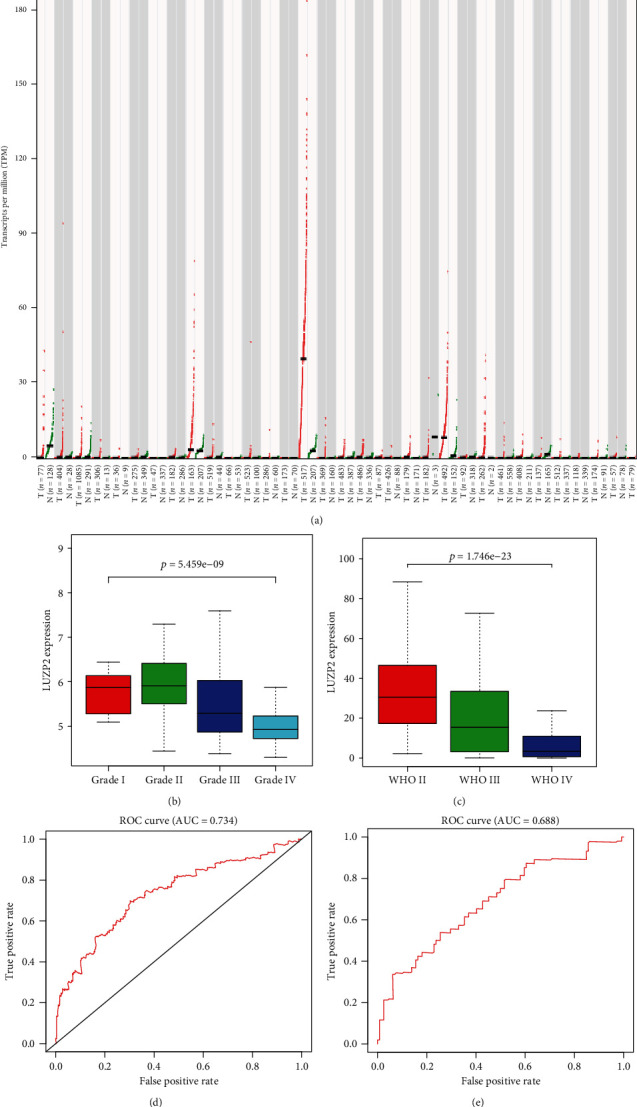
LUZP2 expression profile at the mRNA level in the normal and different grade glioma tissues. (a) LUZP2 mRNA expression in different normal human tissues and tumor tissues. LUZP2 mRNA expression in different grade glioma tissues in (b) CGGA and (c) GSE16011. Validation of LUZP2 overexpression for HCC prediction using the ROC curve in (d) TCGA cohort and (e) CGGA cohort.

Using the GEPIA mRNA-seq data of various tumors and normal tissues, we found that the expression of LUZP2 in normal tissues was slightly high in the adrenal gland, brain, and prostate and low in others. In LGG, adrenocortical carcinoma, and prostate cancer tissues, the expression of LUZP2 was higher than that of matched normal tissues (Figure 1(a)). Figures 1(b) and 1(c) show that the expression of LUZP2 decreased with the increase of tumor grade in the GSE16011 and CGGA part C cohort. The AUC value of LUZP2 expression for LGG diagnosis was 0.734 in TCGA-LGG cohort (Figure 1(d)) and 0.688 in the validation part C cohort (Figure 1(e)). These results suggest that LUZP2 was significantly upregulated at the mRNA level in glioma tissues and may have diagnostic significance in the clinic. However, the expression of the LUZP2 mRNA level decreased gradually with the increase of tumor grade.

### 3.2. LUZP2 Expression Was Significantly Correlated with LGG Subtypes

**Figure 2:**
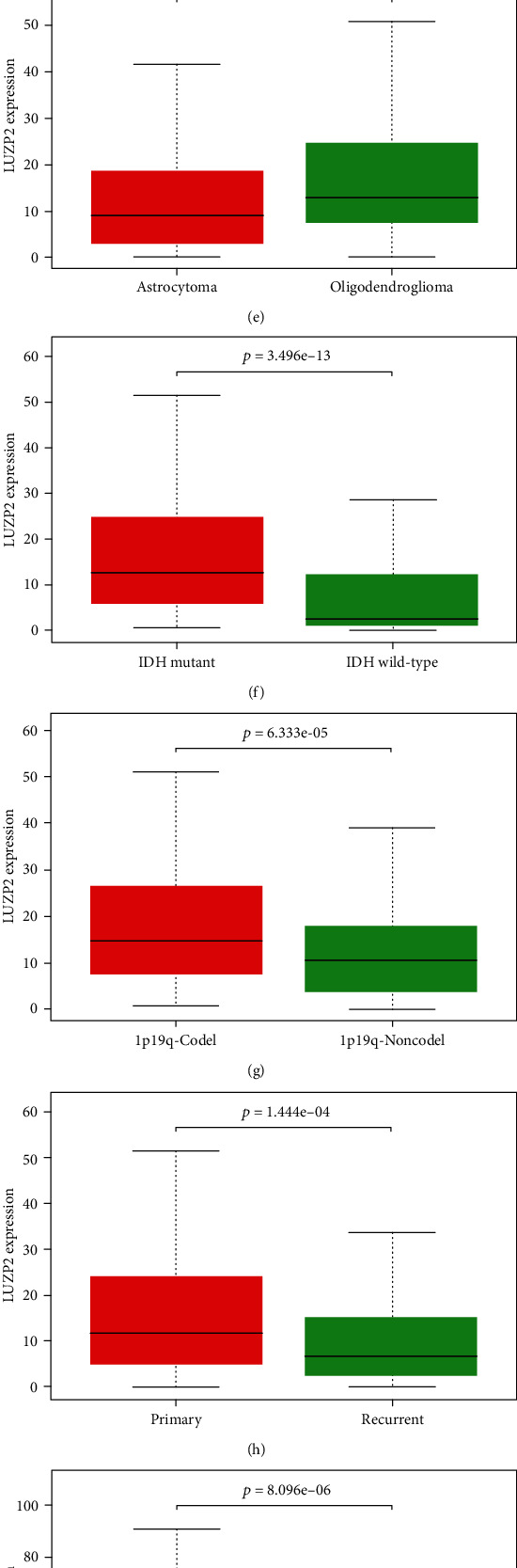
LUZP2 expression profile at the mRNA level by clinical characteristics in TCGA and validation cohort. (a) Grade in TCGA. (b) Histology in TCGA. (c) IDH1 mutant status in TCGA. (d) Grade in CGGA. (e) Histology in CGGA part B. (f) IDH1 mutant status in CGGA part B. (g) 1p19q codeletion in CGGA part B. (h) Tissue type in CGGA part B. (i) Grade in CGGA part C. (j) Histology in CGGA part C. (k) IDH1 mutant status in CGGA part C. (l) Tissue type in CGGA part C.

In view of the significant decrease of LUZP2 with the increase of glioma grade, we further study the relationship between LUZP2 and tumor subtypes. Through TCGA and CGGA part B and part C dataset, we explored the relationship between LUZP2 and glioma grade, histology classification, IDH mutant status, 1p19q codeletion status, and whether the tumor is a recurrent sample. We found that for grade III gliomas, IDH wild-type gliomas, 1p19q noncodeletion gliomas, and recurrent gliomas, LUZP2 had a lower expression level (Figure 2: all *p* < 0.05). However, the relationship between LUZP2 and glioma histology is still uncertain. The above results showed that the expression of LUZP2 decreased significantly with the increase of tumor malignancy.

### 3.3. Low LUZP2 Expression Independently Predicted Poor OS in LGG

**Figure 3:**
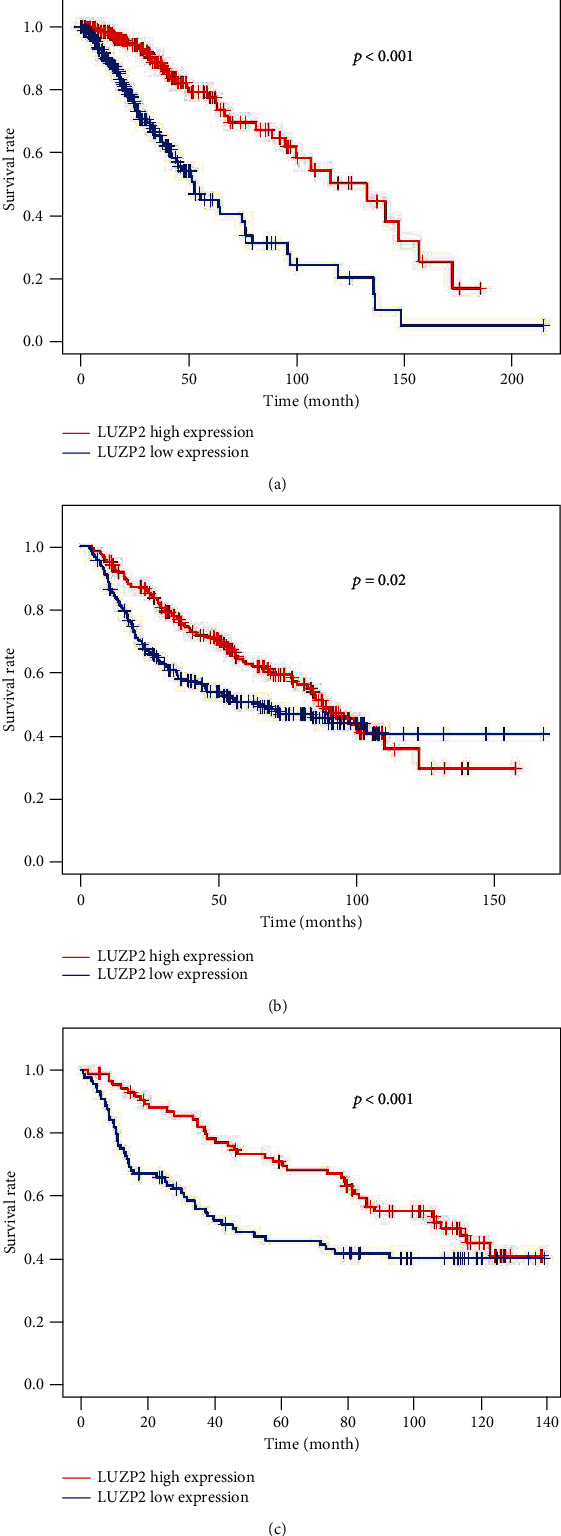
Kaplan-Meier survival analysis by median. (a) Kaplan-Meier curves of overall survival in TCGA. (b) Kaplan-Meier curves of overall survival in the CGGA part B dataset. (c) Kaplan-Meier curves of overall survival in the CGGA part C dataset.

We then estimated the prognostic value of LUZP2 in LGG patients. Baseline patient characteristics from TCGA and CGGA are shown in Table S1. Kaplan-Meier analysis indicated that low LUZP2 expression was associated with poor OS in TCGA-LGG cohort and validation cohort (Figure 3). In the univariate Cox analysis in TCGA cohort, characteristics, such as age, grade, IDH1 mutant status, radiotherapy, histology, and LUZP2 expression, were associated with survival. The multivariate Cox analysis indicated that age, grade, histology, and LUZP2 expression were prognostic factors that could predict LGG patient overall survival independently. In the CGGA part B dataset, only grade, histology, and LUZP2 expression were statistically significant. Oddly enough, only gender, grade, and 1p19q codeletion status have statistical significance in the CGGA part C dataset. The results of the univariate and multivariate Cox analyses in TCGA and CGGA LGG cohorts are listed in Tables 1–3.

### 3.4. Construction of a Transcription Factor Regulatory Network

**Figure 4:**
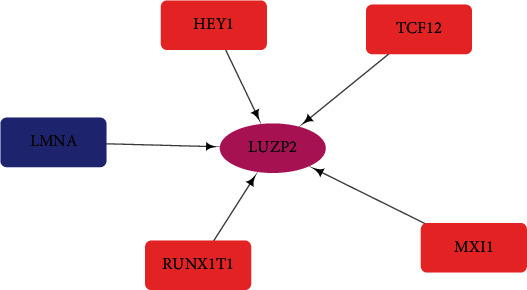
Regulatory network of TFGs and LUZP2. Red indicates that the overexpression of the TFG can upregulate LUZP2, while blue indicates that the overexpression of the TFG can downregulate LUZP2. The thicker the line, the higher the correlation coefficient.

To explore the possible mechanism of LUZP2 dysregulation in LGG, we analyzed the correlation between TFGs and LUZP2 expression. Five TFGs meet the criteria of *p* value below 0.001 and correlation coefficient greater than 0.4. Among them, HEY1, TCF12, MXI1, and RUNX1T1 were positively correlated with LUZP2 expression, indicating that their high expression may promote the expression of LUZP2. LMNA was negatively correlated with LUZP2 expression, suggesting that its high expression may inhibit LUZP2 expression. In order to describe the regulation relationship more clearly, we constructed a regulation network based on TFGs, as shown in Figure 4.

### 3.5. miR-142-5p Upregulation Contributed to LUZP2 Downregulation in LGG

**Figure 5:**
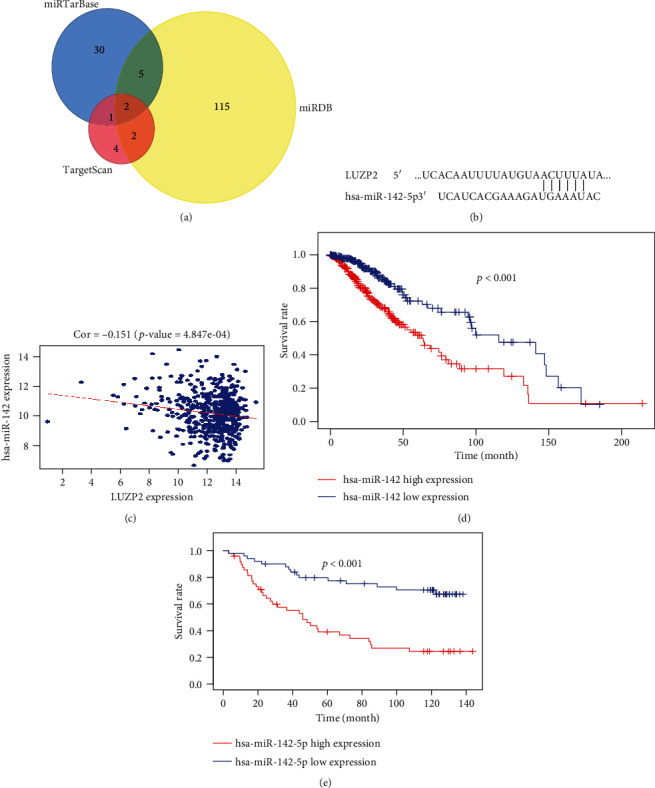
LUZP2 is targeted by miR-142-5p in LGG. (a) Selection of potential regulatory miRNAs of LUZP2 in 3 miRNA-mRNA interaction prediction and validation website. (b) The putative binding site of LUZP2 3′UTR by miR-142-5p. (c) Correlation between miR-142-5p and LUZP2 mRNA expression. (d, e) Kaplan-Meier curves of overall survival of miR-142-5p in TCGA and CGGA.

The potential regulatory miRNAs of LUZP2 were obtained from the predicted databases miRDB and TargetScan and experimentally supported database miRTarBase. miR-5590-3p and miR-142-5p are found in the Venn diagram (Figure 5(a)). Since miR-5590-3p is not significantly expressed in TCGA and CGGA miRNA databases, miR-142-5p was finally screened as a candidate for further analysis and validation. Linear regression analyses indicated that the miR-142-5p expression level was negatively correlated with LUZP2 mRNA expression (Pearson's *R* = −0.151, *p* = 0.0004847) (Figure 5(c)). Furthermore, Kaplan-Meier analysis revealed that high miR-142-5p expression was correlated with poor OS in TCGA-LGG (*p* < 0.001) and validation cohort (*p* < 0.001) (Figures 5(d) and 5(e)). This indicates that high miR-142-5p expression may be involved in the downregulation of LUZP2.

### 3.6. Coexpression Analysis and Functional Enrichment Analysis

**Figure 6:**
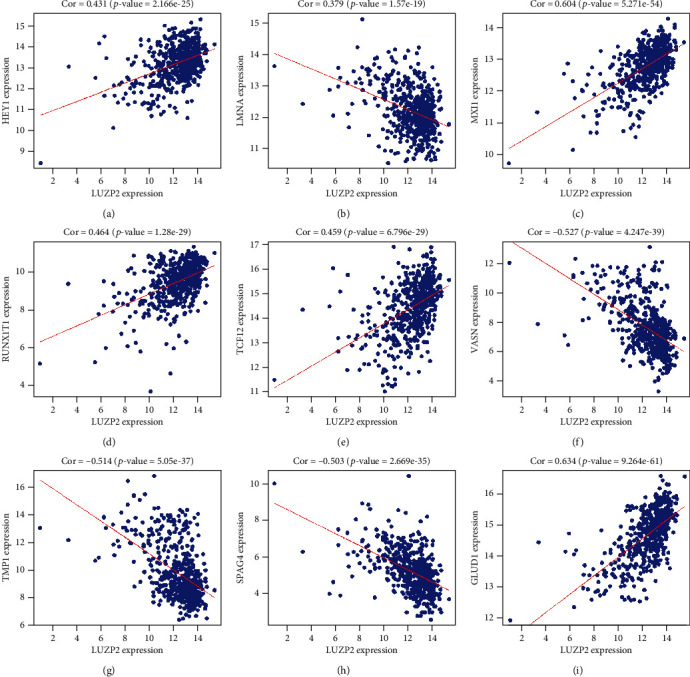
9 selected genes correlated to LUZP2 in TCGA.

**Figure 7:**
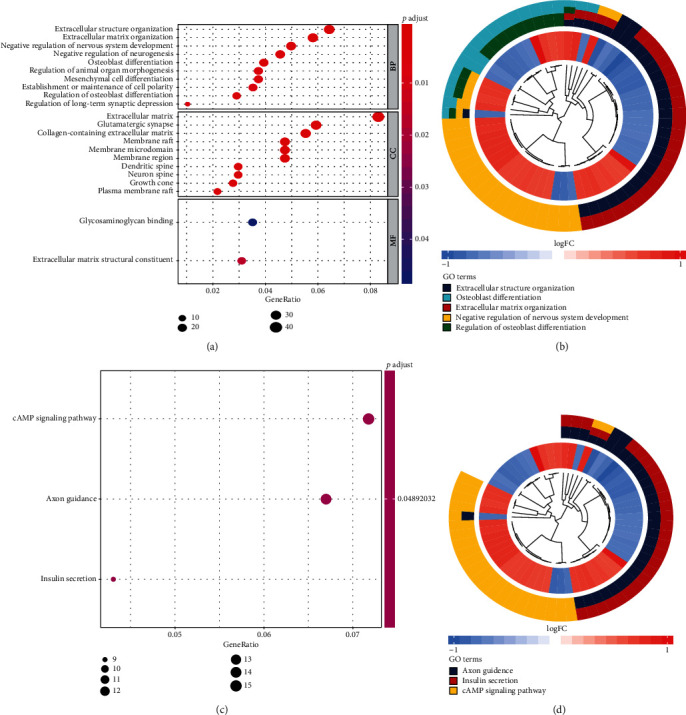
Gene ontology and pathway enrichment analysis of coexpression genes. (a, b) GO term analysis. (c, d) KEGG pathway analysis.

568 coexpression genes were identified via the expression of LUZP2 in TCGA-LGG cohort. The coexpressive relationship of the five transcription factors described above and the other four genes most significantly associated with LUZP2 are shown in Figure 6. The coexpression genes were categorized into the BP, CC, and MF functional groups in GO terms. The coexpression genes in the BP group were mainly enriched in extracellular structure organization, extracellular matrix organization, negative regulation of nervous system development, and negative regulation of neurogenesis. The genes in the CC group were significantly enriched in the extracellular matrix, glutamatergic synapse, collagen-containing extracellular matrix, dendritic spine, and neuron spine. The genes in the MF group were mainly enriched in the extracellular matrix structural constituent and glycosaminoglycan binding (Figure 7). From the Kyoto Encyclopedia of Genes and Genomes (KEGG) pathway analysis, we demonstrated that these genes were mainly involved in the axon guidance, insulin secretion, and cAMP signaling pathway. These results indicated that the coexpression genes were mainly involved in nervous system extracellular matrix development and transduction of signals in the cell surface.

## 4. Discussion

In recent years, the development of bioinformatics has greatly changed the biomedical research. Discovering the hub gene and verifying its role in experiments have been applied in many articles and show great value [19]. Identifying hub biomarkers can help improve tumor diagnosis, judge the prognosis, and personalize the treatment. The application of IDH, which has been written into the 2016 WHO Classification of Tumors of the CNS, shows the great significance of molecular diagnosis in gliomas [3]. However, the current clinical application of molecular markers is far from meeting the needs. More molecular biomarkers need to be developed urgently.

LUZP2, as a protein specifically expressed in the nervous system, has rarely been studied before. Through GEPIA and the expression profile of LUZP2 in TCGA, CGGA, and GSE16011, we found that, as in prostate cancer, the expression of LUZP2 mRNA in gliomas was upregulated compared with normal brain tissue but downregulated during the development from low to high grade [12]. This indicates that the decrease of LUZP2 may be an important sign of gliomas transforming from low grade to high grade.

Furthermore, we explored the clinical significance of LUZP2 expression in LGG. The low expression of LUZP2 was closely related to grade III gliomas, IDH wild-type gliomas, 1p19q noncodeletion gliomas, and recurrent gliomas. The grade III gliomas, IDH wild-type gliomas, 1p19q noncodeletion gliomas, and recurrent gliomas were all the poor prognostic factors of LGG patients. Taken together, these results showed that the expression of LUZP2 decreased significantly with the increase of tumor malignancy. Therefore, we have reason to believe that LUZP2 is a good indicator of tumor malignancy. In the process of glioma production and increased malignancy, the acquisition, loss, and reacquisition of LUZP2 are interesting. It is necessary to investigate potential mechanisms in the future.

The present study further explored the predictive ability of LUZP2 on the prognosis of glioma. K-M analyses showed that low expression of LUZP2 was associated with unfavorable OS (*p* < 0.001). Based on the univariate and multivariate analyses in TCGA dataset, the mRNA expression of LUZP2 was an independent prognostic factor (*p* < 0.001) with age, grade, and histology. The same trends appeared in the CGGA part B dataset (*p* < 0.05). These findings indicated that the LUZP2 expression level served as a valuable prognostic indicator in LGG. But in the CGGA part C dataset, LUZP2 was no longer an independent prognostic factor. This may be due to the small sample size and different components. Furthermore, LUZP2 is closely related to independent prognostic factors such as grade and IDH, which makes it not significant in multivariate Cox analysis. More datasets are needed to verify the exact role of LUZP2 in glioma.

The transcription factors are important regulators of gene expression and play an important role in the development of tumors. In order to explore the regulation mechanism of LUZP2, we examined the correlation between transcription factor expression and LUZP2. We found that 5 transcription factors were highly correlated with LUZP2 expression. HEY1, TCF12, MXI1, and RUNX1T1 were positively correlated with LUZP2 expression, indicating that their high expression may promote the expression of LUZP2. LMNA was negatively correlated with LUZP2 expression, suggesting that its high expression may inhibit LUZP2 expression. Through the GEPIA website [13], we could find that in TCGA-LGG database, the expression of TCF12, MXI1, and RUNX1T1 was positively correlated with the prognosis of LGG. LMNA was negatively correlated with the prognosis of LGG, while HEY1 is not significantly correlated with the prognosis. Of these TFGs, the role of TCF12, RUNX1T1, and LMNA in gliomas is still unclear and needs further investigation. MXI1 acted as a tumor suppressor in human glioblastomas involving the transcriptional downregulation of cyclin B1 gene expression [20]. Through this study, it is possible that MXI1 can also suppress the glioma through LUZP2 which is still to be explored. HEY1 promotes maintenance of neuronal precursor cells and glial versus neuronal fate specification [21]. The role of HEY1 in gliomas may be very closely related to its effect on LUZP2. The TF regulatory network will lay the foundation for future research on the mechanism of action of LUZP2 in gliomas.

miRNAs which can downregulate target genes by inducing mRNA degradation and translation obstruction through binding the target mRNA can also be the key regulators of gene expression [22]. Through three databases, we eventually got miR-142-5p, which is one of the genes most negatively correlated with LUZP2 by the coexpression analysis (*p* < 0.001). The role of miR-142-5p in the development of malignant tumors has been widely reported, including colorectal cancer [23, 24], which can promote the progress of cancer, and gastric cancer [25], which can inhibit the metastasis of cancer. In glioma, miR-142-5p is reported to suppress the stem cell-like traits of glioma cells, but its effect on LGG overall survival is still unclear [26]. This study indicated that in patients with LGG, high miR-142-5p expression means low survival, which needs to be further confirmed by multivariate analysis. Considering the putative binding site of LUZP2 3′UTR by miR-142-5p, we speculate that miR-142-5p may regulate the expression of LUZP2 negatively through competing endogenous RNAs in the upstream of LUZP2, which needs further experimental support.

In the body, the function of molecules is often not alone but through the interaction with other molecules. And they often have a complex regulatory network relationship. The most common regulatory network is the regulation of expression. Molecules with the same expression pattern are often closely related in function. This makes it possible to explore the role of a positional gene through a common expression pattern. Since the role of LUZP2 is unknown, we searched for genes with the same expression pattern through coexpression analysis. These genes may be an important part of the LUZP2 regulatory network, and the biological function of LUZP2 may be closely related to them. In GO analysis, BP, CC, and MF are all enriched in extracellular matrix development-related pathways and transduction of signals. From the Human Protein Atlas (HPA) database, we know that LUZP2 is mainly located in the soma and synapse in neurons, and it can be secreted out from neurons [27]. It has been reported that LUZP2 is a positive modulator of neuroendocrine differentiation [28], and the possible role of the LUZP2 in glioma may be related to it. Combined with the role of LUZP2 coexpression genes in GO analysis, we make a bold speculation that LUZP2 in glioma can be an important regulator involved in the regulation of neuroendocrine function and development of extracellular matrix. The expression of LUZP2 in the nervous system-specific table was disordered, the neuroendocrine function decreased, and the tumor cells were not like nerve cells but closer to stem cells. When the tumor level has not increased, LUZP2 has begun to decline and the prognosis has begun to deteriorate. From the analysis of the correlation between the molecular expression and the clinical subtypes, we could find that LUZP2 of IDH mutant cells decreased, which indicates that the neuroendocrine function of IDH mutant glioma cells is worse, which may be related to the role of IDH. Others such as glioma grade and 1p19 codeletion status are the same. When the tumor grade has not increased, LUZP2 has begun to decline and the prognosis has begun to deteriorate. We think it is very important to judge the prognosis of tumors by the secretory function of tumor cells.

Due to the lack of specific interventions, merely bioinformatics analysis cannot replace cell and in vivo experiments. Although the interaction between miR-142-5p and LUZP2 has been found in the miRTarBase dataset, it is still unclear whether they have any interaction in gliomas. What we need to do next is to overexpress and knock down miR-142-5p in the glioma cell line to verify the regulatory effect of miR-142-5p on LUZP2 and then select the genes significantly correlated with LUZP2 in coexpression analysis to detect the changes of the upstream and downstream genes and biological effects. Overall, our analysis emphasized the importance of LUZP2 in low-grade glioma and established a complete regulatory network from transcription factors to miRNA to downstream signaling pathways, which provided great convenience for the in vitro intervention experiment. Of course, the lack of experimental intervention is a major drawback of our article.

## 5. Conclusions

In conclusion, low expression of LUZP2 independently predicted a poor prognosis in LGG patients. miR-142-5p upregulation could be the upstream regulator that contributed to LUZP2 downregulation. LUZP2 may be crucial for nervous system extracellular matrix development and serve as an important clinical biomarker for LGG patients. Additional studies are necessary to further confirm the exact role of LUZP2 in LGG.

## Figures and Tables

**Table 1:** Cox proportional hazards regression model analysis of overall survival in TCGA.

Variable	Univariate analysis		Multivariate analysis
	Wald	*p*	*p*
LUZP2 expression (high vs. low)	35.268	<0.001	<0.001
Gender (male vs. female)	0.347	0.556	
Race	2.343	0.310	
Karnofsky score (>80 vs. ≤80)	0.499	0.480	
Age group	55.105	<0.001	<0.001
Grade (III vs. II)	42.688	<0.001	<0.001
Histology	9.747	<0.01	<0.001
IDH1 mutant status (yes vs. no)	14.49	0.001	0.140
Radiotherapy (yes vs. no)	21.049	<0.001	0.067

**Table 2:** Cox proportional hazards regression model analysis of overall survival in CGGA part B.

Variable	Univariate analysis		Multivariate analysis
	Wald	*p*	*p*
LUZP2 expression	11.029	0.001	0.047
Gender (male vs. female)	0.213	0.645	
Age	0.734	0.291	
Grade (II vs. III)	37.623	<0.001	<0.001
Histology	34.323	<0.001	0.074
IDH1 mutant status (yes vs. no)	19.332	<0.001	0.124
1p19q codeletion status (yes vs. no)	27.873	<0.001	0.514
Radiotherapy (yes vs. no)	3.007	0.083	0.372
Chemotherapy (yes vs. no)	0.480	0.488	
MGMT	1.607	0.205	

**Table 3:** Cox proportional hazards regression model analysis of overall survival in CGGA part C.

Variable	Univariate analysis		Multivariate analysis
	Wald	*p*	*p*
LUZP2 expression	6.014	0.014	0.365
Gender (male vs. female)	5.448	<0.05	0.048
Age group	17.639	0.001	0.221
Grade (II vs. III)	41.3	<0.001	<0.001
Histology	22.039	<0.001	0.073
IDH1 mutant status (yes vs. no)	19.987	<0.001	0.891
1p19q codeletion status (yes vs. no)	45.630	<0.001	0.002
Radiotherapy (yes vs. no)	4.351	0.037	0.150
Chemotherapy (yes vs. no)	14.805	0.001	0.116

## Data Availability

The datasets used during the present study are available from the corresponding author upon reasonable request.

## Abbreviations

PC: Prostate cancer
LGG: Low-grade glioma
TCGA: The Cancer Genome Atlas
GEO: Gene Expression Omnibus
CGGA: Chinese Glioma Genome Atlas (CGGA)
OS: Overall survival
GO: Gene Ontology
KEGG: Kyoto Encyclopedia of Genes and Genomes
ROC: Receiver operating characteristic
AUC: Area under the curve value
TFGs: Transcription factor genes
HPA: Human Protein Atlas.

## Supplementary Materials

Supplementary Materials Table S1: baseline patient characteristics of included observations from TCGA and CGGA.
