# Supplementary material for: Downregulation of LUZP2 Is Correlated with Poor Prognosis of Low-Grade Glioma
Source: Biomed Res Int. 2020 Jul 9;2020:9716720. doi: 10.1155/2020/9716720 (PMC7368956; doi:10.1155/2020/9716720)
Supplement: Supplementary Materials — Table S1: baseline patient characteristics of included observations from TCGA and CGGA. [file 9716720.f1.pdf]

Table 1. Baseline Patient Characteristics of Included Observations from TCGA and CGGA.

Variable	TCGA cohort	CGGA cohort1	CGGA cohort2
**Gender**			
Male	279(55.2)	182(44.0)	66(37.9)
Female	226(44.8)	232(56.0)	108(62.1)
**Race**			
White	465(92.1)		
Black	21(4.2)		
Other/unknown	19(3.8)		
**Age group**			
<31	96(19.0)	78(18.8)	24(13.8)
31-40	152(30.1)	131(31.6)	77(44.3)
41-50	103(20.4)	142(34.3)	46(26.4)
>50	154(30.5)	63(15.2)	27(15.5)
**Grade**			
WHO II	245(48.5)	171(41.3)	100(57.5)
WHO III	260(51.5)	243(58.7)	74(42.5)
**Histology**			
Astrocytoma	191(37.8)	248(59.9)	64(36.8)
Mix glioma	128（25.3）	29(7.0)	73(42.0)
Oligodendroglioma	186（36.8）	137(33.1)	37(21.3)
**IDH1_mutant**			
Wildtype	34(6.7)	90(21.7)	44(25.3)
Mutant	91(18.0)	286(69.1)	129(74.1)
Unknown	380(75.2)	38(9.2)	1(0.6)
**1p19q_codeletion_status**			
Codel		125(30.2)	57(32.9)
Non-codel		252(60.9)	115(66.1)
Unknown		37(8.9)	2(1.1)
**Chemotherapy**			
YES		278(67.1)	82(47.1)
NO		126(30.4)	82(47.1)
Unknown		10(2.4)	10(5.7)
**Radiotherapy**			
YES	272(53.9)	305(73.7)	20(11.5)
NO	167(33.1)	96(23.2)	149(85.6)
Unknown	66(13.1)	13(3.1)	5(2.9)
**MGMT**			
YES		197(47.6)	
NO		126(30.4)	
Unknown		91(22.0)	
